# Effective Oral Delivery of Teriparatide Using Organoclay—Polymethacrylate Nanocomposites for Osteoporosis Therapy

**DOI:** 10.3390/pharmaceutics17111450

**Published:** 2025-11-10

**Authors:** Gyu Lin Kim, Yeon Ju Kang, Soo Hwa Seo, Jiwoon Jeon, Hyo-Kyung Han

**Affiliations:** College of Pharmacy, Dongguk University, Dongguk-ro-32, Ilsan-Donggu, Goyang 10326, Republic of Korea

**Keywords:** oral formulation, nanocarrier, bone formation, bone resorption, teriparatide

## Abstract

**Background**: Although teriparatide is efficacious, its once-daily subcutaneous injections cause local adverse events, inconvenience, and higher cost, limiting long-term adherence. Therefore, this research aims to engineer a pH-responsive oral formulation of teriparatide for osteoporosis therapy. **Methods**: A layered silicate nanocomplex was obtained by spontaneous self-assembly of teriparatide (Teri) with 3-aminopropyl magnesium phyllosilicate (AC). The nanocomplex (AC-Teri) was then coated with a 1:1 blend of two polymethacrylic acid derivatives (Eudragit^®^ L100 and Eudragit^®^ S 100) to provide pH-triggered drug release along the gastrointestinal tract. **Results**: AC-Teri and the coated nanocomplex (EE/AC-Teri) displayed high encapsulation efficiency (>90%) with narrow size distributions. In a stepwise buffer transition system, EE/AC-Teri demonstrated pH-dependent release, with less than 25% drug liberated at pH 1.2, approximately 54% at pH 6.8, and 74% at pH 7.4 over 24 h. Particle size and ζ-potential of EE/AC-Teri shifted in parallel with dissolution of the outer polymer shell. EE/AC-Teri also protected the peptide against enzymatic degradation, preserving the secondary structure of encapsulated teriparatide in simulated intestinal fluids. Compared with free drug, EE/AC-Teri enhanced transcellular drug permeation 2.7-fold in Caco-2 cells. In dexamethasone-induced osteoporotic rats, oral EE/AC-Teri significantly stimulated bone formation while suppressing resorption; micro-CT and histology confirmed recovery of trabecular architecture. **Conclusions**: EE/AC-Teri represents a promising oral teriparatide formulation for the effective management of osteoporosis.

## 1. Introduction

Osteoporosis is a common skeletal disorder characterized by reduced bone mineral density resulting from excessive resorption or insufficient formation [1]. The disease predisposes patients to hip, wrist, and vertebral fractures. Contributing factors include estrogen deficiency, hepatic disease, and impaired gastrointestinal nutrient absorption [2,3]. Globally, osteoporosis affects over 200 million women, resulting in approximately 8.9 million fractures each year —one every three seconds [4]. Various treatments are available to manage osteoporosis, with focus on slowing bone loss and lowering fracture risk. The most widely prescribed drugs are antiresorptive agents, including bisphosphonates, estrogen, and calcitonin [5]. These agents increase bone mineral density and preserve microarchitecture by inhibiting osteoclastic resorption, but they mainly prevent further deterioration rather than promote new bone formation [6].

By contrast, teriparatide, an anabolic fragment of human parathyroid hormone (hPTH1–34), directly stimulates osteoblast activity and supports new bone formation [7,8,9]. Teriparatide is administered as once-daily subcutaneous injections, which elicit a net increase in bone mass through intermittent stimulation of bone modeling and remodeling [10,11]. However, repeated injections can provoke local reactions—itching, redness, swelling, and infection—along with pain, needle phobia, and higher treatment costs, all of which undermine adherence [12]. Consequently, patients favor oral bisphosphonates over anabolic therapy [13]. Therefore, route of administration is a critical determinant of compliance and therapeutic choice.

Alternative delivery approaches are being actively explored to circumvent these limitations. Among these, oral formulations are attractive since they are safe, painless, convenient, and easy to use, thereby improving patient adherence. However, developing oral delivery systems for teriparatide is highly challenging due to the peptide’s instability in the gastrointestinal tract, poor permeability, and short plasma half-life, all of which result in extremely low bioavailability. Formulation approaches should resolve these issues while ensuring precise pharmacokinetic control. In addition, achieving high encapsulation efficiency, reproducibility, and scalability poses significant technical difficulties. Recently, emerging technologies—including nanoparticle-based carriers and bioengineered oral platforms—seek to increase the gastrointestinal stability and absorption of teriparatide [14,15,16,17]. In a Phase 1 study, the RT-102 robotic pill achieved threefold higher bioavailability than subcutaneous injection and produced no device-related adverse events [16]. Likewise, a Phase 2 trial of an oral rhPTH (1–31)NH_2_ tablet yielded sustained, pulsatile exposure with reproducible pharmacokinetics during a 24-week course [17]. Collectively, these advances could improve the convenience and therapeutic impact of osteoporosis treatment. However, few oral teriparatide products have reached clinical development, and the field still lacks formulations that are stable, scalable, and cost-effective.

Organoclay-based nanoparticles gain a great deal of attention as oral delivery platforms due to their ability to spontaneously self-assemble with therapeutic agents and form organic–inorganic nanocomplexes that enhance structural stability and cellular uptake of the payload [18,19,20,21,22]. In particular, 3-aminopropyl-functionalized phyllosilicate (aminoclay, AC) comprises a lamellar silicate framework covalently grafted with aminopropyl groups [18]. The surface amines impart a net positive charge in aqueous media, enabling electrostatic complexation with anionic drugs such as peptides and nucleotides [22,23]. This nanoconfinement shields cargo from stressors—including elevated temperature and extreme pH. In addition, AC facilitates transmembrane drug uptake via endocytosis and enhances paracellular transport of the payload via a transient tight junction opening [19,21,24,25,26,27,28]. Consequently, AC represents a promising oral carrier for peptide and protein therapeutics that are otherwise prone to enzymatic degradation and show poor intestinal permeability [22]. Coating AC nanocomplexes with pH-dependent polymers further allows controlled-drug release along the gastrointestinal tract [19,21,28]. Among pH dependent polymers, polymethacrylic acid derivatives such as Eudragit L100 and Eudragit S 100, dissolve at pH values above 6.0 and 7.0, respectively [29,30]. Therefore, the surface coating of nanoparticles with these polymethacrylates may prevent the premature drug release and degradation in the stomach, releasing the payload in the intestine where absorption occurs.

**Figure 1:**
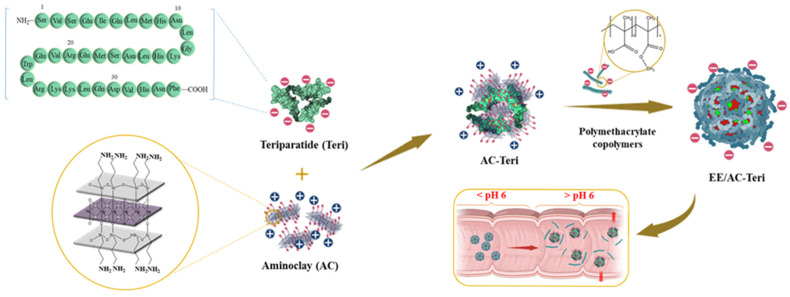
Oral formulation of teriparatide using organoclay/polymethacrylate nanocomposites.

In this study, a teriparatide-loaded AC nanocomplex was fabricated and subsequently coated with polymethacrylic acid derivatives to shield the drug from gastric degradation and enable pH-triggered, gradual drug release throughout the GI tract (Figure 1). The feasibility of this oral formulation as an alternative to subcutaneous teriparatide injection was investigated through integrated in vitro/in vivo studies.

## 2. Materials and Methods

### 2.1. Materials

Teriparatide was purchased from Cold Spring Biotech Co., Ltd. (Guangzhou, China). Pepsin, trypsin, and dexamethasone sodium phosphate were obtained from Sigma-Aldrich Co. (St Louis, MO, USA). Aminoclay (AC) was prepared following a previously reported procedures [20]. Polymethacrylate copolymers were provided by Evonik Korea Ltd. (Seoul, Republic of Korea). For cell culture, Dulbecco’s Modified Eagle’s Medium, Hank’s Balanced Salt Solution (HBSS), fetal bovine serum, antibiotics, and other related reagents were procured from GE Healthcare Life Sciences (South Logan, UT, USA). 3-(4,5-dimethylthiazol-2-yl)-5-(3-carboxymethoxyphenyl)-2-(4-sulfophenyl)-2H-tetrazolium (MTS) assay kit was obtained from Abcam PLC (Cambridge, UK). Caco-2 (human colorectal adenocarcinoma cells, KCLB Cat# 30037.1, RRID:CVCL_0025) and Saos-2 (human osteosarcoma cells, KCLB Cat# 30085, RRID:CVCL_0548) were purchased from the Korean Cell Line Bank (Seoul, Republic of Korea).

### 2.2. Preparation of AC-Based Nanocomplex

After dissolving teriparatide (10 mg/mL) in deionized water, the pH of drug solution was elevated to 11 using 0.5 M sodium hydroxide. This drug solution was then slowly added to an aqueous solution of aminoclay (AC, 10 mg/mL) under stirring, maintaining a 1:1 weight ratio between teriparatide and the clay. After 30 min of mixing, the mixture was centrifuged and the collected white precipitate was subsequently dried under vacuum at an ambient temperature.

The dried nanocomplex (AC-Teri, 30 mg) was dispersed in deionized water (3 mL) and added dropwise into 1% ethanolic solution (6 mL) of polymethacrylates (an equal weight blend (1;1, *w*/*w*) of Eudragit L100 and Eudragit S 100). After 30 min of vigorous stirring, the polymer-coated nanoparticles (EE/AC-Teri) were collected by centrifugation and dried in *vacuo* at room temperature.

### 2.3. Structural Characterization

Size and surface charge of the nanoparticles were assessed using dynamic light scattering (DLS) with a Nano-ZS90 Zetasizer (Malvern Instruments, Malvern, UK). The polydispersity index (PDI), representing the breadth of size distribution, was simultaneously measured and reported as a dimensionless parameter. Encapsulation efficiency (EE) was assessed as follows:

(1) $$\mathrm{EE}(\%)\;=\;\frac{\mathrm{Initial}\;\mathrm{amount}\;\mathrm{of}\;\mathrm{teriparatide}-\mathrm{Amount}\;\mathrm{of}\;\mathrm{free}\;\mathrm{teriparatide}\;\mathrm{in}\;\mathrm{supernatant}}{\mathrm{Initial}\;\mathrm{amount}\;\mathrm{of}\;\mathrm{teriparatide}}\;\times \;100$$

To evaluate the secondary structure of teriparatide, circular dichroism (CD) spectroscopy was performed over 200–260 nm (path length, 0.5 nm). Fourier transform infrared (FT-IR) spectra were collected over the range of 4000–500 cm^−1^. Nanoparticle morphology was visualized by transmission electron microscopy (JEM-F200, JEOL Ltd., Tokyo, Japan).

### 2.4. In Vitro Drug Release Studies

Teriparatide release from AC-Teri and EE/AC-Teri was evaluated under stepwise pH conditions (1.2→6.8→7.4) that emulate gastrointestinal transit [31]. Formulations containing 3 mg of teriparatide, were initially suspended in 75 mL of 0.1 M hydrochloric acid buffer (pH 1.2) and agitated at 100 rpm for 2 h. To simulate intestinal transition, 25 mL of 0.2 M tribasic sodium phosphate buffer was subsequently introduced, adjusting the pH to 6.8, followed by an additional 4 h of stirring. Finally, the medium was titrated to pH 7.4 by adding 0.5 M sodium hydroxide and maintained under constant stirring for 18 h. At predetermined times, 0.2 mL samples were withdrawn and replaced with fresh medium. After filtration of the collected samples with 0.45 µm syringe filters, teriparatide concentration was determined by high-performance liquid chromatography (HPLC). In parallel, real-time monitoring of size and surface charge of EE/AC-Teri was conducted during drug dissolution studies.

### 2.5. Gastrointestinal Stability

The conformational integrity of teriparatide encapsulated within nanocomplexes was assessed under simulated gastrointestinal conditions using enzyme-supplemented media. Specifically, samples were incubated in simulated gastric fluid (SGF; pH 1.2, containing 5 μg/mL pepsin) or simulated intestinal fluid (SIF; pH 7.4, supplemented with 20 μg/mL trypsin) [21]. Nanoparticles corresponding to 0.5 mg/mL of teriparatide were dispersed in 1 mL of either SGF or SIF and incubated at 37 °C with stirring at 100 rpm. Digestion was quenched with 0.2 mL of 0.2 M NaOH (for SGF) or 0.1 M HCl (for SIF) at predefined times. The nanoparticles were then isolated via centrifugation, redispersed in PBS (pH 7.4), and incubated for 2 h. The conformation of teriparatide following its release from nanoparticles was analyzed by CD spectroscopy to verify structural stability.

### 2.6. MTS Assay

The cytotoxic effects of free Teri, AC-Teri, and EE/AC-Teri were assessed using Caco-2 and Saos-2 cell lines. Cells were seeded in 96-well plates at 1 × 10^4^ cells/well. After 24 h-incubation at 37 °C, cells were treated with varying concentrations of teriparatide (either free teriparatide or teriparatide-loaded nanoparticles) for 24 h. Then, MTS reagent (20 µL) was added to each well according to the manufacturer’s protocol, followed by a 3 h incubation. Absorbance was subsequently recorded at 490 nm to determine cell viability relative to untreated controls.

### 2.7. Transport Studies

Caco-2 cells (2.0 × 10^5^ cells/well) were seeded on Transwell^®^ inserts (1.12 cm^2^, 12-well format) and cultured at 37 °C for 21–24 days. The integrity of the epithelial barrier was routinely monitored by measuring transepithelial electrical resistance (TEER). On the study day, residual medium was removed, and cell layers were rinsed twice with PBS (pH 7.4). Hank’s balanced salt solution (HBSS, pH 7.4) was added to the apical (0.5 mL) and basolateral (1.5 mL) chambers. The plates were then equilibrated for 30 min at 37 °C to stabilize the monolayer prior to drug application. Subsequently, 0.5 mL HBSS containing free teriparatide or teriparatide-loaded nanoparticles (final drug concentration 200 µg/mL) was placed in the apical compartment. At predetermined times, samples (100 µL) were withdrawn from the basolateral side and replaced with fresh HBSS. Teriparatide concentrations were quantified by HPLC. TEER was also recorded before dosing, during transport, and after the final wash to monitor barrier integrity. The apparent permeability coefficient (P_app_) was determined as: P_app_ = (dQ/dt)/(A × C_0_), where dQ/dt is the transport rate (µg/s), A is the membrane area (cm^2^), and C_0_ is the initial apical drug concentration (µg/mL).

### 2.8. Efficacy Studies in Dexamethasone-Induced Osteoporosis Rats

The therapeutic effect of orally delivered EE/AC-Teri was examined in dexamethasone-induced osteoporosis rats under an Institutional Animal Care and Use committee (IACUC)-approved protocol (IACUC-2023-039-2). Male Sprague–Dawley rats (280–300 g) were randomly allocated to four groups (*n* = 6 each). The control group received a daily subcutaneous saline. In the osteoporotic model group, 0.1 mg/kg dexamethasone was administered subcutaneously each day to induce bone loss. The injectable teriparatide treatment group was given a combination of daily subcutaneous dexamethasone (0.1 mg/kg) and teriparatide (0.04 mg/kg). The final group was administered EE/AC-Teri orally (16 mg/kg of teriparatide), in combination with daily subcutaneous dexamethasone (0.1 mg/kg). After 8 week-treatment, blood was drawn for biochemical analysis. Blood (0.6 mL) was collected once on day 57 via the jugular vein under 1.5% isoflurane anesthesia in a Tabletop Anesthesia System (Harvard Apparatus, Cambridge, MA, USA). Serum osteocalcin (OCN), procollagen type I N-terminal propeptide (PINP), C-terminal telopeptide of type I collagen (CTx), tartrate-resistant acid phosphatase 5b (TRAP5b), and calcium were quantified with commercial ELISA kits (Thermo Fisher Scientific, Waltham, MA, USA) according to the manufacturer’s instructions. At study completion, animals were euthanized using a 30%/min displacement of chamber air with compressed CO_2_. Both femora were excised and fixed in 4% neutral-buffered formalin for 48 h. Fixed bones were processed for histological and structural analysis. Some samples were paraffin-embedded and sectioned for histological examination using hematoxylin and eosin (H&E) staining. The remaining specimens were subjected to structural evaluation via micro-computed tomography (micro-CT; the Korea Basic Science Institute). Images were acquired using a 90 kV/80 μA X-ray source over a 4 min acquisition time, with the scan field confined to 10 mm. Reconstructed images were generated at an isotropic voxel resolution of 20 μm. Quantitative parameters describing trabecular bone microarchitecture were assessed using Analyze software (ver 12.0; AnalyzeDirect, Overland Park, KS, USA).

### 2.9. HPLC Assay

Teriparatide was quantified by reverse-phase HPLC. Separations were performed on a C18 column (4.6 × 150 mm, 5 μm). The mobile phases were acetonitrile (A) and deionized water (B), each containing 0.1% trifluoroacetic acid. A time-dependent solvent gradient was executed at 1.0 mL/min, starting from 80% B and decreasing to 60% over 2.5 min. This was followed by a rapid transition to 50% over the next 0.5 min, which was sustained for an additional minute. The proportion of solvent B was then elevated back to 60% over 2 min and finally returned to 80% within 3 min. Eluates were detected at 215 nm. Calibration standards (1–200 μg/mL) yielded a linear standard curve (*r*^2^ > 0.998).

## 3. Results and Discussion

### 3.1. Fabrication and Characterization of Nanoparticles

Teriparatide and aminoclay spontaneously self-assembled into a nanocomplex (AC-Teri) via electrostatic attraction between the cationic silicate layers and the anionic peptide, eliminating the need for chemical cross-linkers or harsh processing. AC-Teri exhibited an average hydrodynamic diameter of 264 ± 8.4 nm and a zeta potential of 8.74 ± 0.19 mV (Table 1) with narrow polydispersity, indicating colloidal uniformity. Particles in this size range are favorable for mucosal transport and cellular uptake. In addition, AC-Teri showed high drug encapsulation efficiency, suggesting the efficient incorporation of teriparatide into the carrier matrix with minimal drug loss during fabrication. To enable pH-responsive release, AC-Teri was coated with polymethacrylate copolymers. This enteric layer protects the payload from gastric acid and dissolves in the intestine to permit gradual drug release. After coating, the diameter increased to 569 ± 13.1 nm and the zeta potential shifted to –23.3 ± 1.96 mV (Table 1), confirming successful deposition of the anionic polymer and its capacity to modulate drug release as a function of pH.

**Figure 2:**
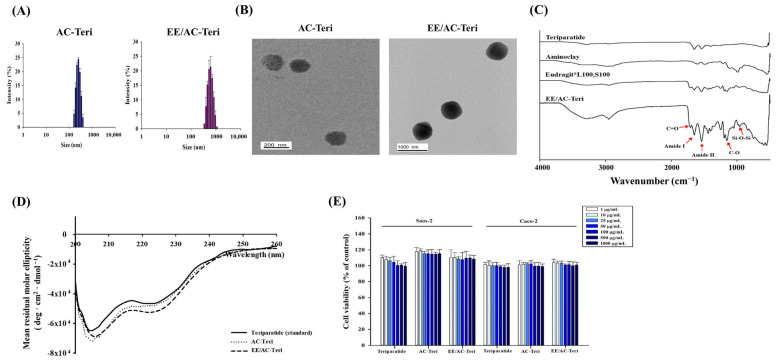
In vitro characterization of teriparatide-loaded nanocomplexes. (**A**) Particle-size distribution (mean ± SD, *n* = 3); (**B**) TEM image; (**C**) FT-IR spectra; (**D**) CD spectra of teriparatide released at pH 7.4; (**E**) Cell viability determined by MTS assay.

TEM revealed that both AC-Teri and EE/AC-Teri were spherical, with diameters consistent with DLS values (Figure 2A,B). FT-IR spectra confirmed that the nanocomplex retained functional groups from teriparatide, aminoclay, and the polymer coating. These characteristic bands indicated that formulation preserved each component’s molecular features without undesired interactions. Specifically, the spectra displayed amide I (1651 cm^−1^) and amide II (1538 cm^−1^) peaks from teriparatide, Si–O–Si (1009 cm^−1^) and Mg–O (550 cm^−1^) vibrations from aminoclay, together with the ester carbonyl (1709 cm^−1^) and C–O stretching (1154 cm^−1^) bands originating from polymethacrylate copolymers (Figure 2C). CD spectroscopy showed that the α-helical structure of teriparatide remained intact after complexation. The CD profile of teriparatide released from AC-based nanoparticles matched that of the native peptide (Figure 2D). Preserving this secondary structure is critical for biological activity and confirms that the fabrication conditions for AC-based nanoparticles are sufficiently mild. Furthermore, drug loaded-nanoparticles did not show any cytotoxicity in Caco-2 and Saos-2 cells (Figure 2E).

In summary, the AC-based nanocomplexes (AC-Teri and EE/AC-Teri) exhibited efficient teriparatide encapsulation with high structural stability.

### 3.2. In Vitro Drug Release Profiles

**Figure 3:**
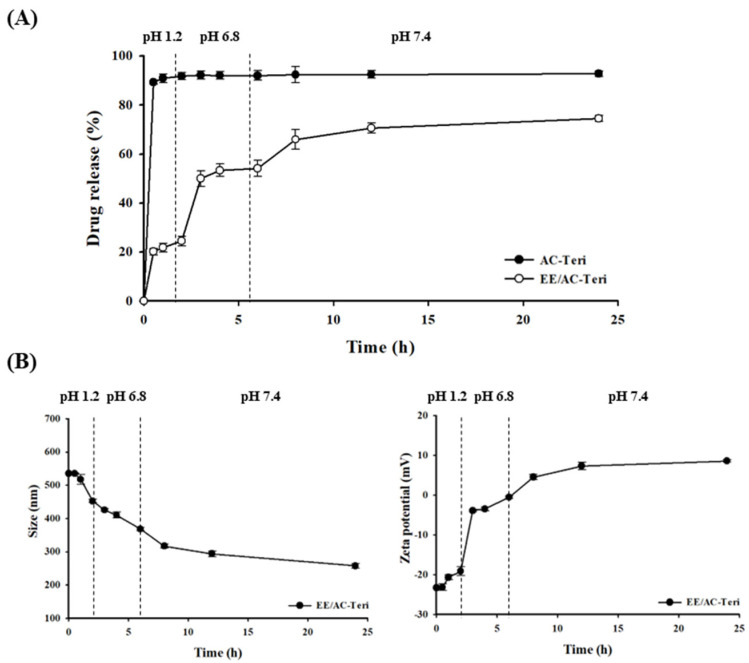
In vitro drug-release profiles of teriparatide-loaded nanocomplexes (mean ± SD, *n* = 3). (**A**) Release from coated (EE/AC-Teri) and uncoated (AC-Teri) nanoparticles during a stepwise pH shift from 1.2 to 7.4. (**B**) Changes in particle size and zeta potential.

To evaluate pH-responsive release, in vitro dissolution studies were conducted using stepwise buffer transitions from pH 1.2 to 6.8 and finally to 7.4, mimicking the progressive pH shifts during gastrointestinal transit. In a strongly acidic medium (pH 1.2), AC-Teri rapidly discharged almost 90% of the encapsulated drug within 30 min (Figure 3A). By contrast, EE/AC-Teri demonstrated a markedly slower and pH-regulated release profile: less than 25% of the drug was liberated under acidic conditions, which then rose to 54% at pH 6.8 and approached 74% at pH 7.4 over 24 h (Figure 3A). Given that two polymethacrylate derivatives used for the surface coating exhibit pH-dependent solubility, the stepwise release reflects progressive dissolution of the enteric coating, which becomes permeable under neutral or alkaline conditions. Supporting physicochemical data (Figure 3B) indicated that EE/AC-Teri retained size and ζ-potential in acid but became smaller and altered its surface charge at pH 6.8–7.4, consistent with polymer dissolution and exposure of the inner complex.

Collectively, EE/AC-Teri affords pH-triggered release, potentially limiting gastric degradation and increasing intestinal teriparatide availability.

### 3.3. Protection Against Enzymatic Degradation

**Figure 4:**
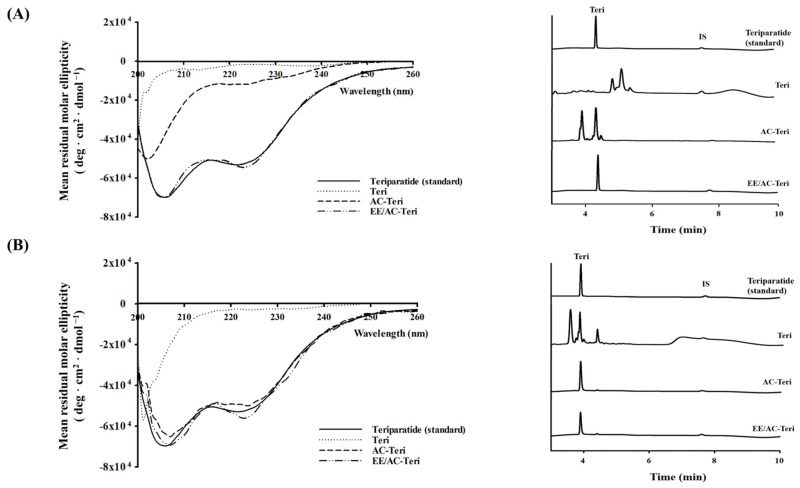
Protection of teriparatide by AC-based nanocomplexes in simulated gastric fluid (SGF) (**A**) and simulated intestinal fluid (SIF) (**B**). (**Left**): CD spectra. (**Right**): HPLC chromatograms.

Proteolytic cleavage by gastric pepsin and intestinal trypsin severely limits oral protein delivery [32]. To evaluate the stability of teriparatide under these harsh conditions, the structural integrity of the drug was examined after exposure to simulated gastrointestinal fluids. Figure 4 shows that free drug underwent pronounced conformational alterations in both SGF and SIF, reflecting the inherent gastrointestinal instability of free peptides. In SIF, AC-Teri was able to preserve the peptide’s structural integrity; however, its protective effect diminished considerably under acidic conditions (Figure 4A,B). In contrast, EE/AC-Teri preserved the native secondary structure of teriparatide in both SGF and SIF (Figure 4A,B). HPLC analysis corroborated these findings by revealing an intact teriparatide peak with no degradation products (Figure 4A,B). These data demonstrate that the enteric-coated nanocomplex shields teriparatide from chemical and enzymatic attack during gastrointestinal transit. This protection obviates chemical modification or protease inhibitors, underscoring EE/AC-Teri’s promise as an oral delivery vehicle.

### 3.4. Cellular Permeability Studies

**Figure 5:**
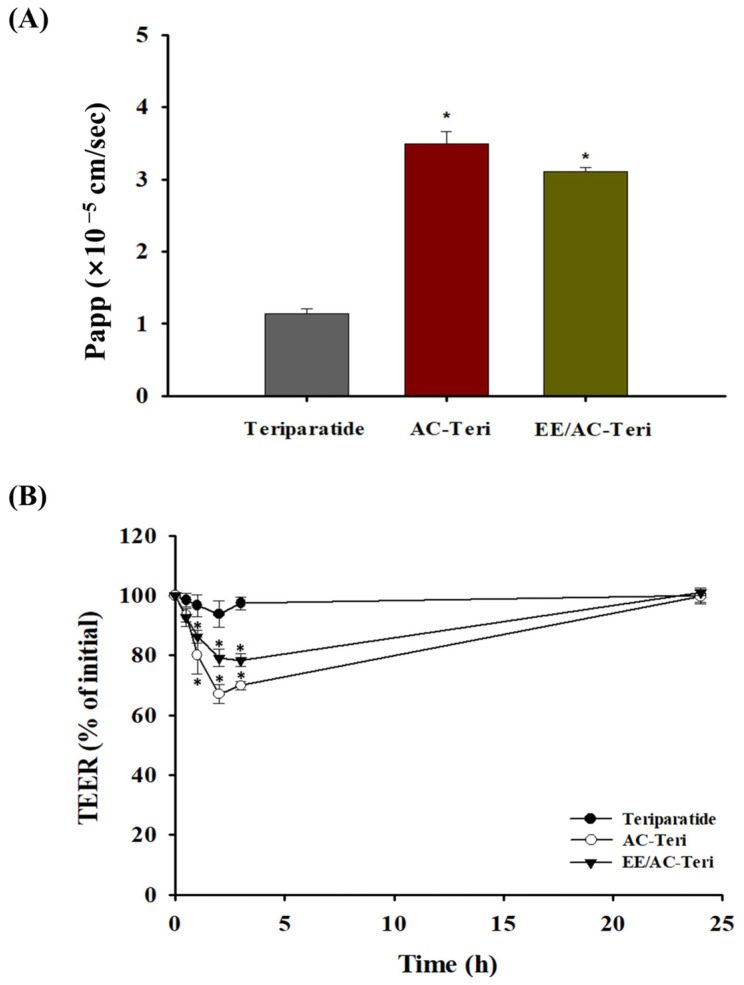
Drug transport across Caco-2 monolayers (mean ± SD, *n* = 6). (**A**) Apparent permeability (Papp) of free teriparatide, AC-Teri, and EE/AC-Teri. (**B**) Transepithelial electrical resistance (TEER) measured in the presence or absence of nanoparticles. * *p* < 0.05 versus free teriparatide.

The intestinal permeability of teriparatide encapsulated in AC-based nanocomplexes was assessed in Caco-2 cells. As illustrated in Figure 5A, AC-Teri and EE/AC-Teri increased transepithelial transport by approximately threefold and 2.7-fold, respectively, relative to free peptide. At pH 7.4, the enteric coating of EE/AC-Teri gradually dissolves; the liberated particles then behave like AC-Teri, yielding a comparable permeability profile (Figure 5A). The higher drug transport achieved with AC-based carriers results most likely from transient tight-junction opening, which enlarges the paracellular route (Figure 5B). In addition, the cationic surface of AC-Teri electrostatically interacts with anionic membrane components, including heparan-sulfate proteoglycans, facilitating endocytic uptake [33]. These complementary mechanisms account for the superior epithelial transport of AC-formulations. These findings highlight the ability of AC-based nanocomplexes to overcome the principal hurdle of oral peptide delivery—low intestinal permeability—without chemical enhancers or protease inhibitors.

### 3.5. In Vivo Efficacy Studies

**Figure 6:**
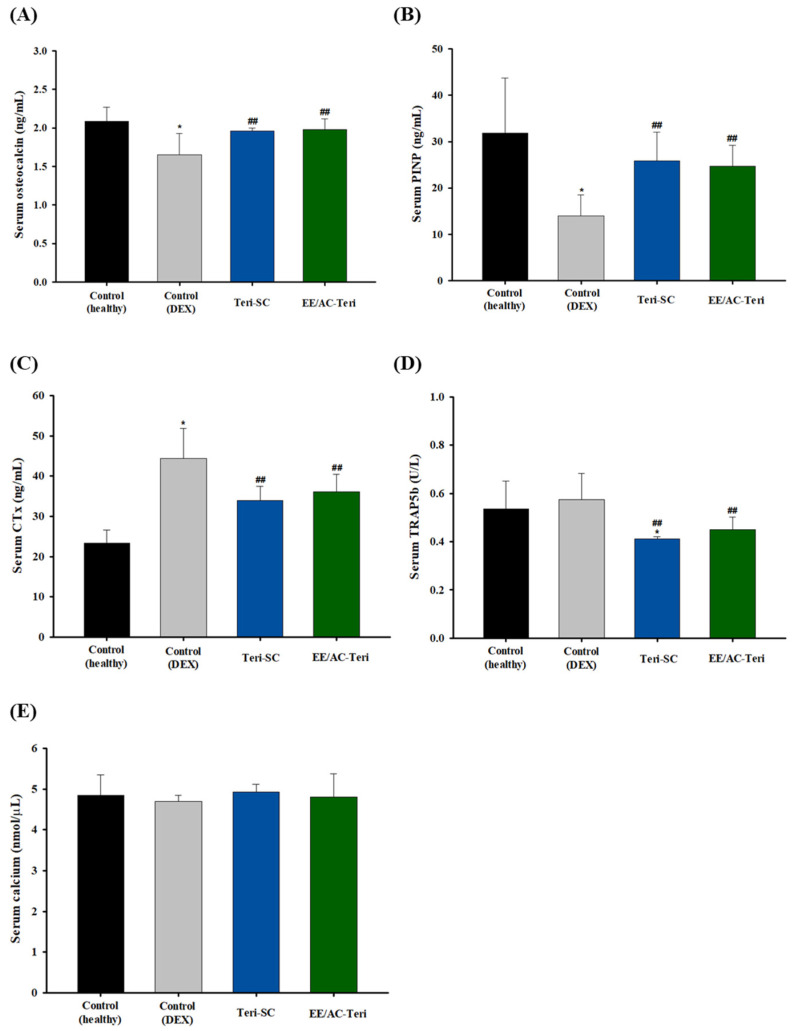
Serum bone-turnover biomarkers after 8 weeks of treatment with oral EE/AC-Teri or subcutaneous teriparatide in dexamethasone-induced osteoporotic rats (mean ± SD, *n* = 6). (**A**) osteocalcin (OCN); (**B**) procollagen type I N-terminal propeptide (PINP); (**C**) C-terminal cross-linked telopeptide of type I collagen (CTx); (**D**) tartrate-resistant acid phosphatase 5b (TRAP5b); (**E**) serum calcium. * *p* < 0.05 versus healthy control; ## *p* < 0.05 versus dexamethasone control.

The therapeutic potential of EE/AC-Teri against glucocorticoid-induced bone loss was investigated in a rat model of dexamethasone-induced osteoporosis. After eight weeks, bone microarchitecture and serum turnover markers were compared with subcutaneous teriparatide. Oral EE/AC-Teri significantly increased the bone-formation markers such as osteocalcin and PINP [34,35] and decreased resorption markers, namely CTx and TRAP5b [36,37] (Figure 6). The resulting metabolic profile favored bone formation and was comparable to that elicited by subcutaneous teriparatide. Serum calcium did not differ between groups, reflecting tight physiological regulation. Glucocorticoids can transiently lower calcium absorption, yet parathyroid calcium-sensing receptors promptly restore homeostasis by stimulating PTH release [38]. Likewise, teriparatide can briefly raise calcium, but levels return to baseline within 16–24 h [39]. Consequently, chronic dosing—either injected or delivered orally via EE/AC-Teri—did not produce sustained hypercalcemia.

**Figure 7:**
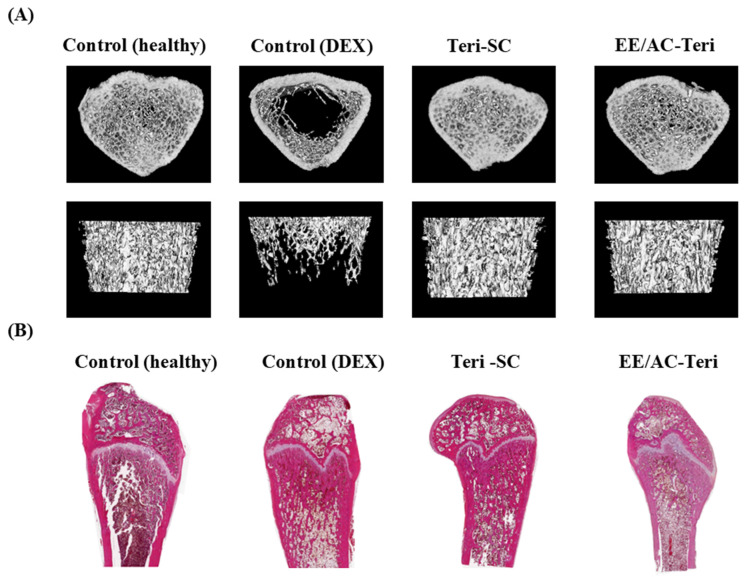
Micro-computed tomography (**A**) and histological examination (**B**) of distal femora from dexamethasone-induced osteoporotic rats after 8 weeks of treatment. Healthy controls received saline; dexamethasone control received 0.1 mg/kg of dexamethasone per day. Drug treatment groups received teriparatide once daily either by subcutaneous injection (0.04 mg/kg) or by oral EE/AC-Teri (equivalent to 16 mg/kg teriparatide), in combination with subcutaneous dexamethasone (0.1 mg/kg).

Micro-CT and histology corroborated the biochemical data (Figure 7). Dexamethasone alone caused severe loss of cancellous bone [40]. Both oral EE/AC-Teri and subcutaneous teriparatide mitigated these lesions, preserving a denser, interconnected trabecular network in the proximal femur (Figure 7A). Histology paralleled the imaging findings (Figure 7B). Healthy rats displayed compact, well-organized trabeculae, whereas dexamethasone-treated controls showed thinned, sparse, and disconnected structures [41]. Subcutaneous teriparatide partially restored architecture, and oral EE/AC-Teri produced similar improvements in trabecular thickness and continuity. Collectively, the results indicate that oral EE/AC-Teri protects bone microstructure as effectively as injectable teriparatide.

**Figure 8:**
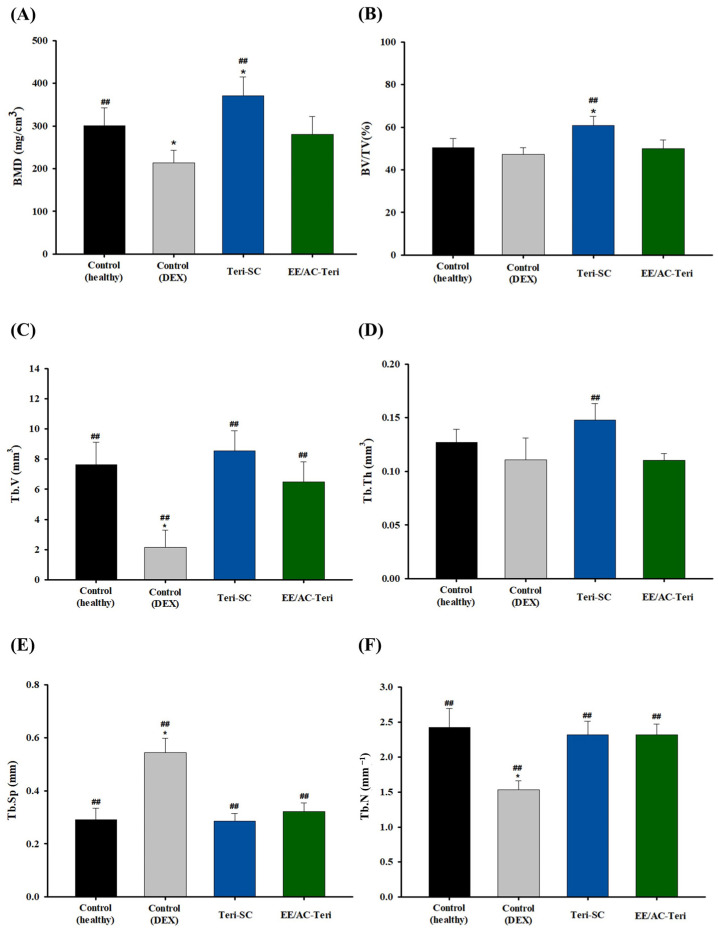
Quantitative micro-computed tomography analysis of trabecular bone (mean ± SD, *n* = 6). (**A**) bone mineral density (BMD); (**B**) bone volume fraction (BV/TV); (**C**) trabecular bone volume (Tb.V); (**D**) trabecular thickness (Tb.Th); (**E**) trabecular separation (Tb.Sp); (**F**) trabecular number (Tb.N). * *p* < 0.05 versus healthy control; ## *p* < 0.05 versus dexamethasone control.

Quantitative parameters obtained from micro-CT analysis are presented in Figure 8. Bone mineral density (BMD), an index of mineral content per unit area or volume, significantly decreased in the Dex-treated group compared with healthy controls, confirming successful induction of osteoporosis [40]. Subcutaneous injection of teriparatide significantly restored BMD and bone volume fraction (BV/TV) relative to the DEX-treated positive control group (Figure 8A,B). Oral EE/AC-Teri also tended to increase BMD, although this improvement did not reach statistical significance. Additional micro-architectural indices [42]—trabecular bone volume (Tb.V), trabecular thickness (Tb.Th), trabecular separation (Tb.Sp), and trabecular number (Tb.N)—were likewise improved by EE/AC-Teri (Figure 8C–F). Particularly, dexamethasone markedly reduced Tb.V and Tb.N, whereas EE/AC-Teri reversed both deficits, suggesting restoration of trabecular bone mass. In addition, EE/AC-Teri markedly decreased Tb.Sp, indicating a denser, more connected trabecular network.

Collectively, the in vivo efficacy studies show that oral EE/AC-Teri improves bone mass and trabecular architecture, highlighting its potential as a non-invasive alternative to subcutaneous teriparatide for the treatment of osteoporosis. Nevertheless, the 400-fold difference between oral and subcutaneous dosing underscores the need for comprehensive pharmacokinetic characterization to clarify the bioavailability of the oral formulation in future studies.

## 4. Conclusions

EE/AC-Teri was developed as an oral formulation of teriparatide, yielding nanoparticles with a mean diameter of 569 ± 13.1 nm and an encapsulation efficiency greater than 90%. The nanocomplex displayed pH-responsive drug release and protected the peptide from enzymatic degradation in simulated intestinal fluid. Encapsulation within the AC matrix increased transepithelial transport across Caco-2 monolayers nearly threefold relative to free teriparatide. In dexamethasone-induced osteoporotic rats, oral EE/AC-Teri stimulated bone formation, elevating serum osteocalcin and PINP, while reducing resorption markers such as CTx and TRAP5b. Micro-CT and histology confirmed the restoration of trabecular microarchitecture and improved bone quality. Overall, oral EE/AC-Teri produced anabolic bone effects comparable to subcutaneous teriparatide, supporting its development as a convenient oral formulation for osteoporosis therapy.

## Figures and Tables

**Table 1:** Characteristics of teriparatide-loaded nanocomplex (mean ± SD, *n* = 3).

Formulation	Size (nm)	PDI	Zeta Potential (mV)	EE (%)
AC-Teri	264 ± 8.4	0.216 ± 0.045	8.74 ± 0.19	90.1 ± 0.3
EE/AC-Teri	569 ± 13.1	0.240 ± 0.071	−23.3 ± 1.96	97.8 ± 1.4

## Data Availability

The raw data supporting the conclusions of this article will be made available by the authors on request.
